# Description of a new Andean species of widow spider (Araneae, Theridiidae, *Latrodectus*)

**DOI:** 10.3897/zookeys.1281.185973

**Published:** 2026-06-02

**Authors:** Jeremy Miller, Chris Alice Kratzer, Charles Griswold

**Affiliations:** 1 Understanding Evolution group, Naturalis Biodiversity Center, Darwinweg 2, 2333 CR Leiden, Netherlands Department of Entomology, California Academy of Sciences San Francisco United States of America; 2 Owlfly LLC: 843 NJ-12 Frenchtown, New Jersey 08825, USA Understanding Evolution group, Naturalis Biodiversity Center Leiden Netherlands; 3 Department of Entomology, California Academy of Sciences, 55 Music Concourse Drive, Golden Gate Park, San Francisco, California 94118, USA Unaffiliated Frenchtown United States of America

**Keywords:** Andes, citizen-science, DNA barcode, iNaturalist, *Latrodectus lucacha*, new species, Peru, species distribution model, taxonomy

## Abstract

We describe a new species of widow spider (Araneae, Theridiidae, *Latrodectus*) from the Andean region. DNA barcode sequences are provided. The species is documented from museum specimens across Peru and identified from photographs on iNaturalist, extending its inferred distribution into Ecuador, Bolivia, and Chile. We integrate specimen and citizen-science occurrences to generate a species distribution model using WorldClim bioclimatic variables, predicting highest suitability along temperate to high-elevation Andean regions. We discuss the utility and limitations of citizen-science imagery for delimiting and mapping species and summarize available information on clinical aspects of envenomation in Peru associated with the local “lucacha” widow spider.

## Introduction

To this day, Herbert W. Levi remains one of the most productive and expert authors of taxonomic publications in the history of spider systematics (Zhang et al. 2025), particularly within the web building families Araneidae, Tetragnathidae, and Theridiidae, with emphasis on the Americas. Levi was a careful observer of anatomical details and recognized that spider genitalia are among the richest sources of characters for distinguishing one species from another (Levi 1965). He was also aware that taxonomic scholarship on the medically important genus *Latrodectus* Walckenaer, 1805 (Theridiidae) was not entirely satisfactory, and that rectifying this situation would not be easy. Levi implemented a rigorous methodological approach that emphasized examination of a large sample of museum specimens from as many places as possible while relying on a series of standard anatomical views to facilitate comparison. He relied on a study of relevant type specimens and legacy literature to help him to make sense of the clusters of similarity he found (Leibensperger 2016). While this time-honored approach produces respectable results for spider taxonomy in most cases, it seems almost uniquely unsuited to *Latrodectus*.

Levi’s global taxonomic revision of the genus *Latrodectus* was published in 1959. Among his principal conclusions was that many of the species recognized as valid at the time were actually synonyms, and that instead of 22 species, there were really only six. Most nominal species worldwide were synonymized under the name *Latrodectus mactans* (Fabricius, 1775), while most species described from South America were circumscribed under *L. curacaviensis* (Müller, 1776). Both widespread species were presented as having considerable intraspecific variation (Levi 1959).

But Levi’s taxonomic schema for *Latrodectus* began to fall apart almost immediately. In the course of researching *Latrodectus* from the Santiago del Estero region of Argentina, J.W. Abalos could not reconcile Levi’s low diversity concept of *Latrodectus* with what he observed in the field (Abalos 1962). The character system that first stood out in this regard was egg-case morphology, something rarely emphasized by museum-based taxonomists. But by studying egg-case morphology in the field, it appeared that at least four distinct sympatric *Latrodectus* species were present in Santiago del Estero. The validity of this unconventional character system was challenged based on variation observed among egg cases constructed by a single individual (Gerschman and Schiapelli 1965). However, a later study suggested that the critique lacked sufficient rigor (Pinter 1967). Developed through field observations and laid down in a series of papers (Abalos 1962; Abalos and Báez 1967; Abalos 1980), each of the four egg-case types they described is now associated with a different valid *Latrodectus* species.

Upon reviewing the new evidence, Levi accepted the new scientific consensus. Commenting forthrightly on his previous work he wrote “for some time it had been apparent that all my conclusions were wrong” (Levi 1983: 195).

Levi maintained a global perspective that was progressive for his time. Herb and Lorna Levi collaborated on the first global study of theridiid genera (Levi and Levi 1962); only George and Elisabeth Peckham had previously done something similar for the global genera of the jumping spider family Salticidae (Peckham and Peckham 1886). Classically, spider taxonomists typically focused on their home country and its colonies, in many cases trying to describe all spiders even if they were expert in none of them. Naturally, people working in such isolation would create synonyms; Levi’s approach was an attempt to rectify this but went too far. Yet in recognizing his error he delivers a lesson that epitomizes the true essence of science as a knowledge system that becomes more robust by learning from its mistakes.

Decades later, Garb et al. (2004) presented the first phylogeny of *Latrodectus* based on COI sequence data. Unfortunately, this analysis also included some taxonomic errors concerning the South American fauna. Several specimens were misidentified, and these errors propagated through several subsequent analyses (Aguilera et al. 2009; Rueda et al. 2021; Valdez-Mondragón and Cabrera-Espinosa 2023); this issue is now being rectified (Miller et al. in prep.).

Garb et al.’s (2004) phylogeny suggested that the species found in North and South America belonged to a monophyletic lineage distinct from the rest of the world. Miller et al. (in prep.) found that the North American and South American lineages represented independent colonization events from Afroeurasia.

Among South American species investigated by Miller et al. (in prep.) was an undescribed species from several sites in Peru. DNA sequences were obtained from several specimens and are included in the phylogenetic study of Miller et al. (in prep.); this is the species we describe here.

## Methods

Photographs were made with a DS-Ri2 digital camera using NIS Elements software. Composite extended focus images were made with Helicon Focus v. 7 (Kozub et al. 2000). Habitus illustrations by Giovanni Maki were rendered on coquille board, then scanned and finished in Adobe Illustrator. The line drawing of female reproductive structures was based on an epigynum cleared in KOH, photographed, and rendered in Adobe Photoshop. Anatomical terminology for the male pedipalp follows Knoflach and van Harten (2002) with further interpretation based on the anatomical studies of Agnarsson (2004), Agnarsson et al. (2007), and Bhatnagar and Rempel (1962). Measurements in millimeters were made using an ocular micrometer. Descriptions refer to red marking on the opisthosoma; these often lose color, fading to yellow or white in ethanol preserved specimens.

Leg muscle tissue in lysis buffer with proteinase K was used as the source material for DNA extraction. Extractions proceeded using the Thermo Scientific KingFisher Flex extraction robot with the Macherey-Nagel NucleoMag 96 Tissue kit at the Naturalis Biodiversity Center lab facility. The standard COI barcoding region (Hebert et al. 2003) was amplified using the primers LCO1490 (5'-GGTCAACAAATCATAAAGATATTGG-3') (Folmer et al. 1994) and Chelicerate Reverse 2 (5'-GGATGGCCAAAAAATCAAAATAAATG-3') (Barrett and Hebert 2005). PCR started with denaturation at 94 °C for 180 s, followed by 40 cycles of 94 °C for 15 s, 50 °C for 30 s, and 72 °C for 40 s, finishing with a 72 °C extension for 300 s. Barcode sequences and associated data were uploaded to the Barcode of Life Data Systems (BOLD; http://www.boldsystems.org/).

**Figure 1.**
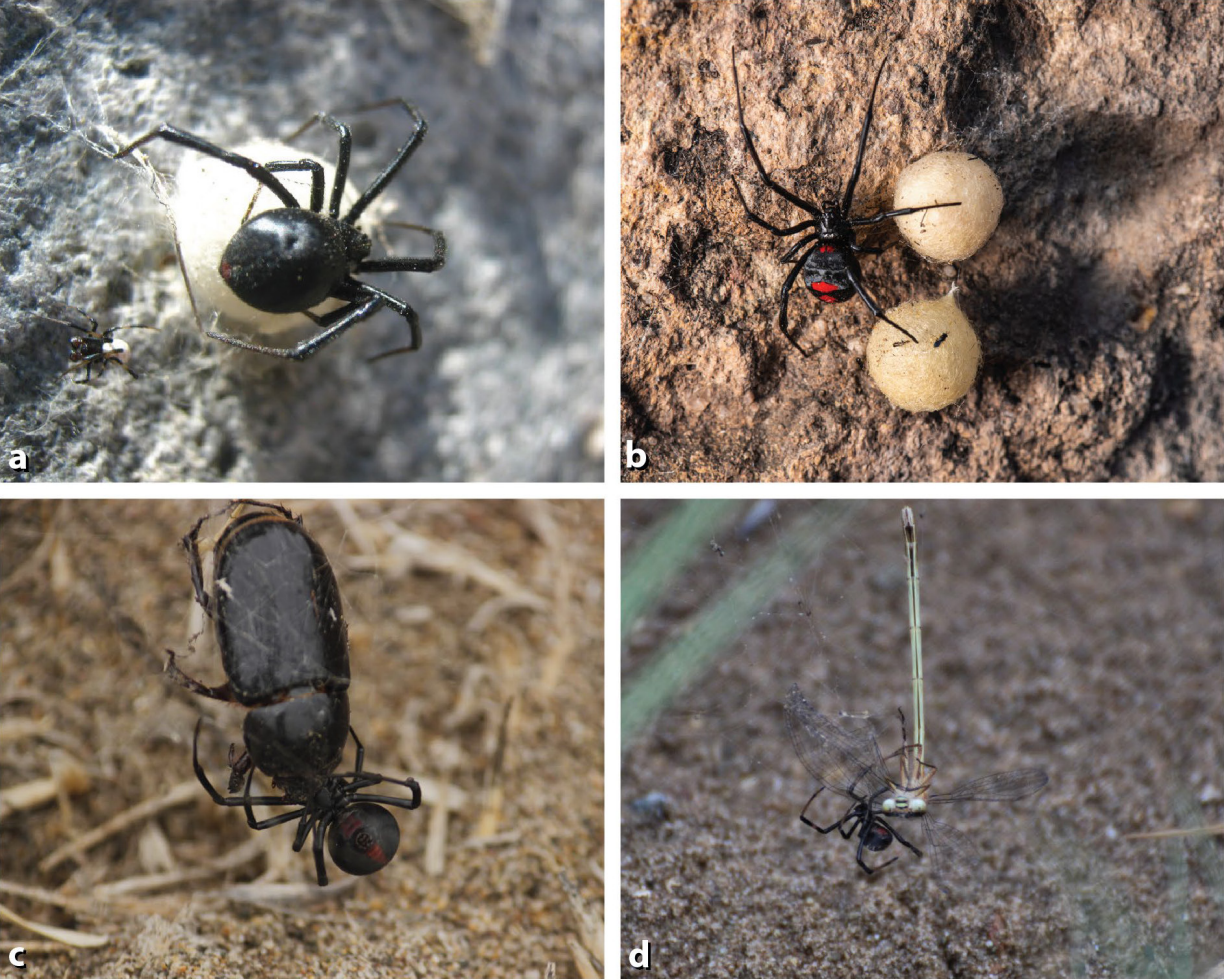
Observations of *Latrodectus lucacha* Miller, Kratzer & Griswold, sp. nov., from iNaturalist. **A**. Female, male and egg case, from Arica, Arica y Parinacota, Chile; https://www.inaturalist.org/observations/61855205 (by ferru, CC-BY-NC); **B**. Female with two egg cases, note parasitoid wasps on egg case, from Nabón, Azuay, Ecuador; https://www.inaturalist.org/observations/183846604 (by kabirbosques, CC-BY); **C**. Female feeding on a beetle, from Lima, Peru; https://www.inaturalist.org/observations/66898772 (by ruthgo, CC0); **D**. Female feeding on a damselfly, from Lima, Peru; https://www.inaturalist.org/observations/171632844 (by Mathieu_fr, CC-BY-NC).

Specimens examined were georeferenced (when necessary) and plotted on a map. Inferred coordinates given in square brackets; verbatim coordinates without brackets. We conducted a survey of iNaturalist records in an effort to better estimate the distribution of this species based on observations from the citizen-science community. We examined ~1,000 iNaturalist observations meeting the following criteria: (1) identified as *Latrodectus* (genus-level or below); (2) observed date on or before 30 April 2025; (3) photographed in Colombia, Ecuador, Peru, Bolivia, or Chile; and (4) licensed for reuse (CC-0, CC-BY, CC-BY-SA, CC-BY-NC, CC-BY-ND, CC-BY-NC-SA, or CC-BY-NC-ND). Records were screened for a diagnostic combination of coloration and egg-case morphology consistent with *L. lucacha* sp. nov., and only records judged identifiable with high confidence were retained (e.g., Fig. 1). Records of specimens examined were combined with the records we identified on iNaturalist to create a species distribution model. All specimens examined for this study are deposited in Museo de Historia Natural (**MUSM**), Lima, Peru.

### Species-distribution model

A species-distribution model was created in MAXENT v. 3.4.4 (Phillips et al. 2025) with data preparation and variable selection conducted in R v. 4.4.2 (R Core Team 2024). Occurrence records were spatially filtered to one record per 5 arc minute grid cell to reduce sampling bias. Environmental data consisted of the 19 standard bioclimatic variables (https://www.worldclim.org/data/bioclim.html) at the 5-minute scale, averaged over 1970–2000, from WorldClim v. 2.1 (Fick and Hijmans 2017). Variables were cropped to the region with occurrence records. To reduce autocorrelation, we examined pairwise Pearson’s correlation coefficients, considering values > 0.7 as correlated. Among the temperature-related variables, only BIO3 (Isothermality) and BIO4 (Temperature Seasonality) were not correlated with Annual Mean Temperature (BIO1). BIO12 (Annual Precipitation) was correlated with all other precipitation variables; BIO15 (Precipitation Seasonality) had the lowest coefficient of variation with BIO12. Based on these comparisons and a Variance Inflation Factor (VIF) test (all < 10), we evaluated two alternative variable sets, both consisting of BIO1, BIO12, and BIO15, and then alternatively including BIO3 or BIO4. Models were run with a background of 10,000 randomly selected points. We used 10 replicates with cross-validation, the default regularization multiplier of 1.0, and all feature classes enabled.

## Results

Both alternative species distribution models performed well (mean test AUC > 0.94) with a slightly better value for the BIO4 model (0.946) compared to the BIO3 model (0.942) (Suppl. material 1: fig. S1A). The omission line is close to predicted, suggesting good calibration without overfitting issues (Suppl. material 1: fig. S1B). Jackknife tests of variable importance showed that Annual Mean Temperature (BIO1) and Annual Precipitation (BIO12) were individually the most informative predictors (AUC of each alone ~0.9). Temperature Seasonality (BIO4) achieved moderate discrimination (AUC ~0.71), whereas Isothermality (BIO3) was weaker (AUC ~0.63); Precipitation Seasonality (BIO15) was intermediate (AUC ~0.67). Thus, the model including BIO4 was used to predict distribution probability (Suppl. materials 1, 2: figs S1C, D, S2).

### Latrodectus lucacha

Taxon classificationAnimaliaAraneaeTheridiidae

Miller, Kratzer & Griswold sp. nov.

1B00E948-59C4-56BD-9C81-8ACD99562886

https://zoobank.org/6D3D9F4C-7F66-4A2A-A57C-92AD510E6B3E

Figs 1, 2, 3, 4, 5, 6, 7, 8

#### Material examined.

***Holotype*: Peru** • ♀; Junin, Pachacayo; 11°46.321'S, 75°42.425'W; 3540 m; August 2006; G. Binford, A. Merrell, M. Weber, W. Paredes, L. Tejada; BOLD: AWSLL004-26; MUSM-ENT 0519569.

***Paratypes*: Peru** • 1♀; Arequipa, Atiquipa; 15°45.675'S, 74°22.20'W; 2665 ft [812 m]; 4 August 2006; G. Binford, A. Merrell, M. Weber, W. Paredes, L. Tejada; BOLD: AWSLL001-26; MUSM-ENT 0519562 • 1♀; Arequipa, Bella Union (km 532); 15°30.061'S, 74°50.815'W; 322 ft [98 m]; 3 August 2006; G. Binford, A. Merrell, M. Weber, W. Paredes, L. Tejada; BOLD: AWSLL002-26; MUSM-ENT 0519564 • 1♂; Arequipa, Socabaya; [16.47°S, 71.54°W]; 2300 m; 23 January 2005; L. Tejada; MUSM-ENT 0519563 • 1♀; Cusco, Chumbivilcas, Pampa Cancha; 14°25'44"S, 71°49'47"W; 3890 m; June 2013; L. Huerto; pasonal y cultivo, pitfalls; BOLD: AWSLL005-26; MUSM-ENT 0519570 • 1♂; Cusco, Chumbivilcas, Pampa Cancha; 14°25'44"S, 71°49'47"W; 3890 m; June 2013; L. Huerto; pasonal y cultivo, pitfalls; BOLD: AWSLL006-26; MUSM-ENT 0519571 • 1♀; La Libertad, Huanchaco; 8°4.467'S, 79°7.068'W; 22 m; 19 August 2006; G. Binford, A. Merrell, M. Weber, W. Paredes, L. Tejada; BOLD: AWSLL003-26; MUSM-ENT 0519565 • 1♀; Lima, Pantanos de Villa Om; [12.215°S, 76.985°W]; 1 March 2005; L. Tejada; on grass in sandy ground near wetlands; BOLD: AWSLL007-26; MUSM-ENT 0519567 • 1♂; Lima, Pantanos de Villa Om; [12.215°S, 76.985°W]; 1 March 2005; L. Tejada; on its web on sand near wetlands; BOLD: AWSLL008-26; MUSM-ENT 0519568 • 1♀; Lima, Pantanos de Villa Om; [12.215°S, 76.985°W]; 1 March 2005; L. Tejada; on grass in sandy ground near wetlands; MUSM-ENT 0519566.

#### Etymology.

Lucacha is a common name for widow spiders in Peru (Pickard-Cambridge 1902; Maguiña Vargas et al. 2017).

#### Diagnosis.

**Figure 2.**
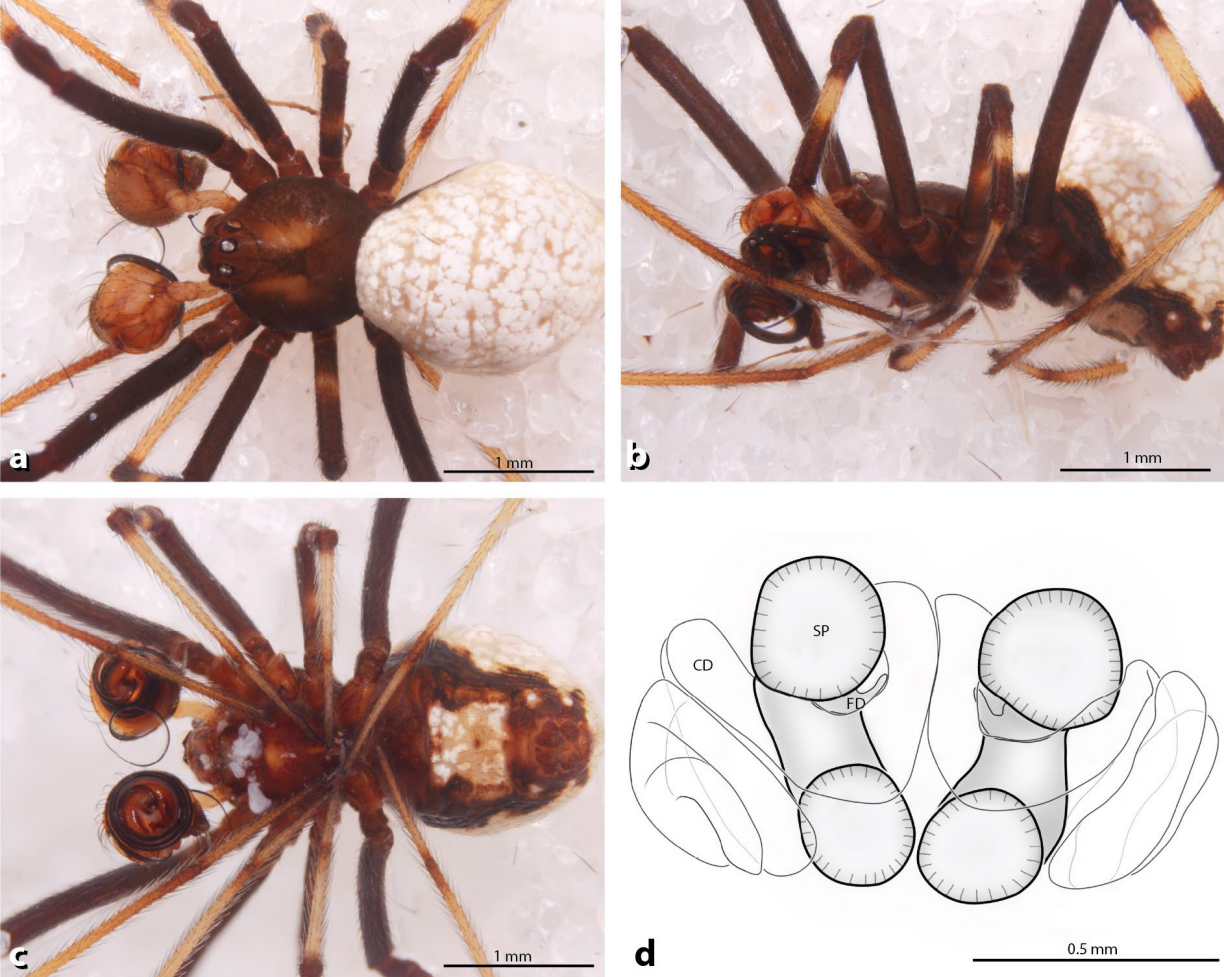
*
Latrodectus lucacha* Miller, Kratzer & Griswold, sp. nov. **A–C**. From Pantanos de Villa Om, Lima, Peru (MUSM-ENT 051958), microphotographs of adult male; **D**. From Pachacayo, Junin, Peru (MUSM-ENT 0519569), schematic illustration of female reproductive structures; **A, D**. Dorsal; **B**. Lateral; **C**. Ventral. Abbreviations: CD copulatory duct, FD Fertilization duct, SP Spermathecae. Scale bars: 1.0 mm (**A–C**), 0.5 mm (**D**).

**Figure 3.**
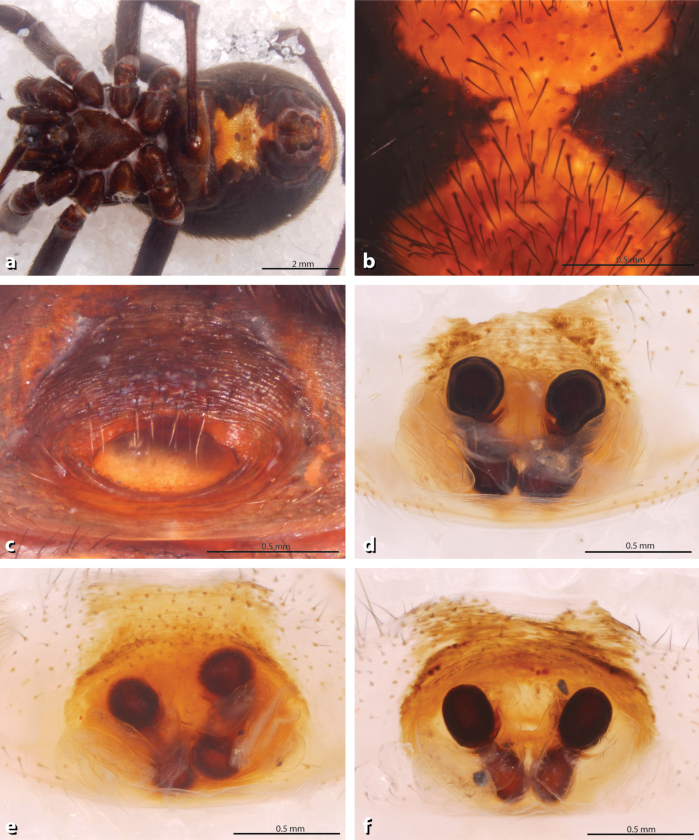
*
Latrodectus lucacha* Miller, Kratzer & Griswold, sp. nov., female reproductive structures and opisthosoma. **A–D**. Holotype from Pachacayo, Junin, Peru (MUSM-ENT 0519569); **E**. From La Libertad, Huanchaco, Peru (MUSM-ENT 0519565); **F**. From Bella Union, Arequipa (MUSM-ENT 0519564); **A**. Adult female, ventral view; **B**. Opisthosomal setae, posterior view; **C**. Epigynum, ventral view; **D–F**. Vulva, dorsal view. Scale bars: 2.0 mm **(A)**, 0.5 mm **(B–F)**.

**Figure 4.**
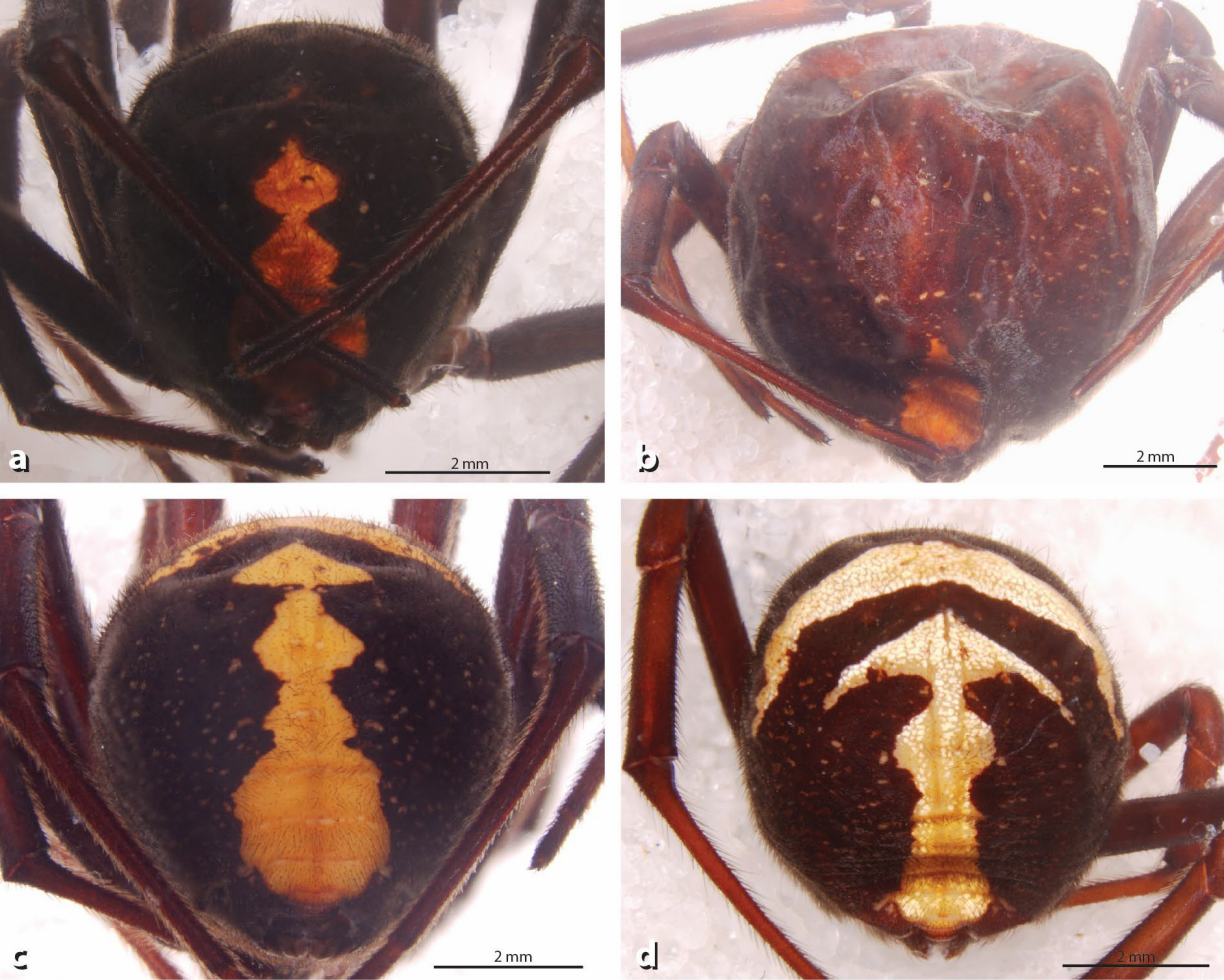
*
Latrodectus lucacha* Miller, Kratzer & Griswold, sp. nov., posterior view of adult female opisthosoma. **A**. Holotype from Pachacayo, Junin, Peru (MUSM-ENT 0519569); **B**. From Atiquipa, Arequipa, Peru (MUSM-ENT 0519562); **C**. From Pampa Cancha, Cusco, Peru (MUSM-ENT 0519570); **D**. From Bella Union (km 532), Arequipa, Peru (MUSM-ENT 0519564). Scale bars: 2.0 mm.

**Figure 5.**
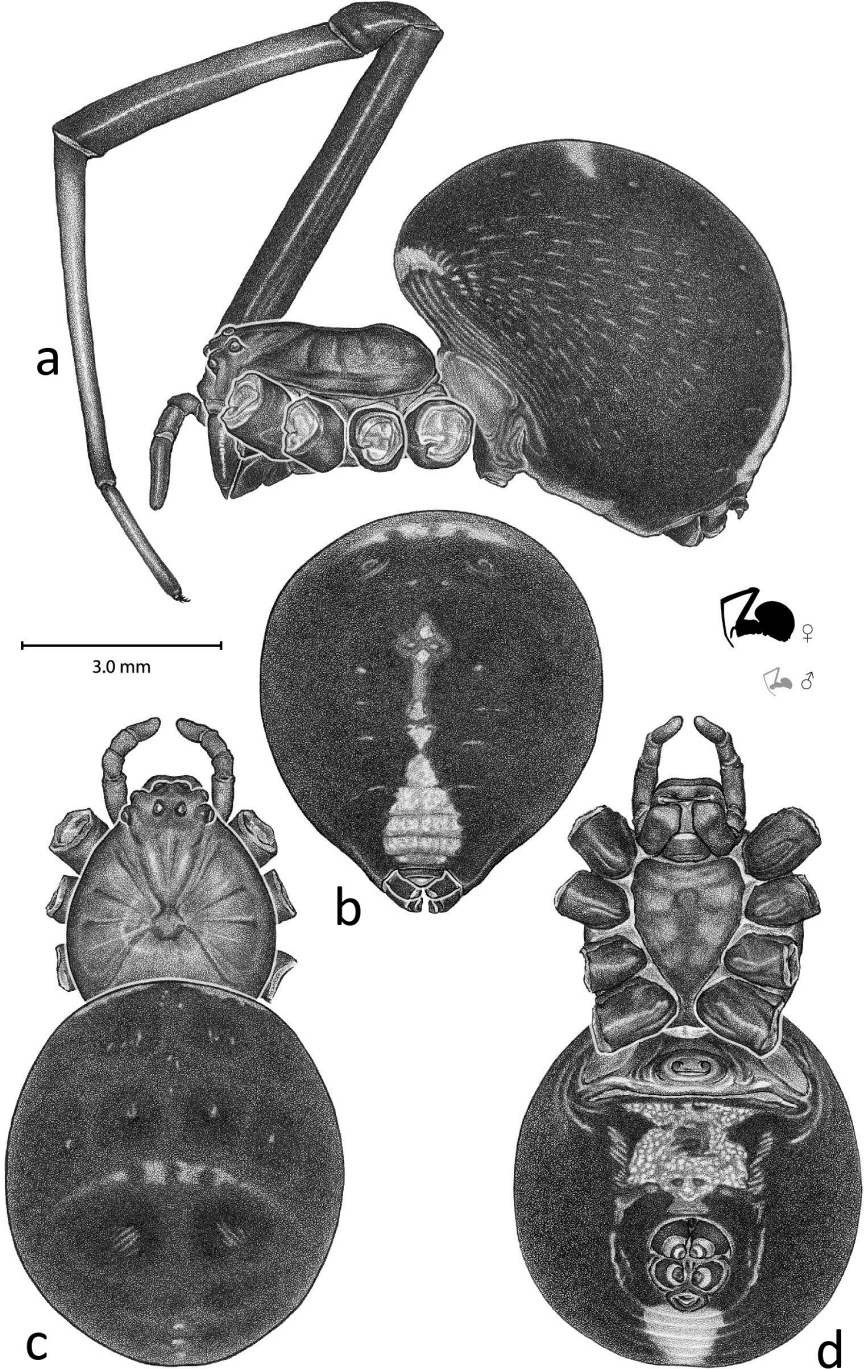
*
Latrodectus lucacha* Miller, Kratzer & Griswold, sp. nov., from Atiquipa, Arequipa, Peru (MUSM-ENT 0519562) illustrations of adult female. **A**. Lateral; **B**. Opisthosoma, posterior; **C**. Dorsal; **D**. Ventral. Inset, silhouettes of female (black) and male (gray) showing relative size. Scale bar: 3.0 mm.

Female with three loop copulatory ducts (Figs 2D, 3D–F), distinguishing it from *L. geometricus* C. L. Koch, 1841 and *L. quartus* Abalos, 1980 (Abalos 1980: figs 39, 66), which have more loops (ca 4), and from *L. antheratus* (Badcock, 1932) and *L. variegatus* Nicolet, 1849 (Abalos 1980: figs 49, 58), which have fewer (2). With dorsal longitudinal opisthosomal stripe variable in both width and length (Figs 4, 5), resembling several South American species, but not exhibiting oblique vertical stripes on the posterior quarter found in *L. antheratus*, *L. corallinus* Abalos, 1980, *L. curacaviensis*, and *L. hurtadoi* Rueda & Realpe, 2021 (Abalos 1980: figs 33, 54, 56; Rueda et al. 2021: figs 3A, 5A); *L. garbae* Rueda & Realpe, 2021 may also have these stripes, but in this species with the most red on the opisthosoma, dorsal markings have largely merged with each other (Rueda et al. 2021: fig. 4A, B). Ventral opisthosomal marking box-like with distinct lateral notches anterior to the center, resembling *L. diaguita* Carcavallo, 1960, *L. variegatus* and *L. thoracicus* (including *L. mirabilis* (Holmberg, 1876), Figs 3A, 5D; Abalos 1980: figs 7, 8, 27, 60), although in the latter species the posterior part of the marking can be lost, leaving just two oblique stripes, resembling *L. quartus* (Abalos 1980: fig. 41); by contrast *L. antheratus*, *L. garbae*, *L. corallinus*, and *L. hurtadoi* have a more or less square mark with a gap near the center (Abalos 1980: fig. 34, 55; Rueda et al. 2021: figs 3C, 4D); *L. curacaviensis* and *L. geometricus* have hourglass-like markings with constrictions near the center (Rueda et al. 2021: figs 2F, 5D, 12A, B). Most South American species including *L. lucacha* sp. nov. have long opisthosomal setae without much variation in length, but *L. diaguita* and *L. quartus* have dimorphic long and short opisthosomal setae.

**Figure 6.**
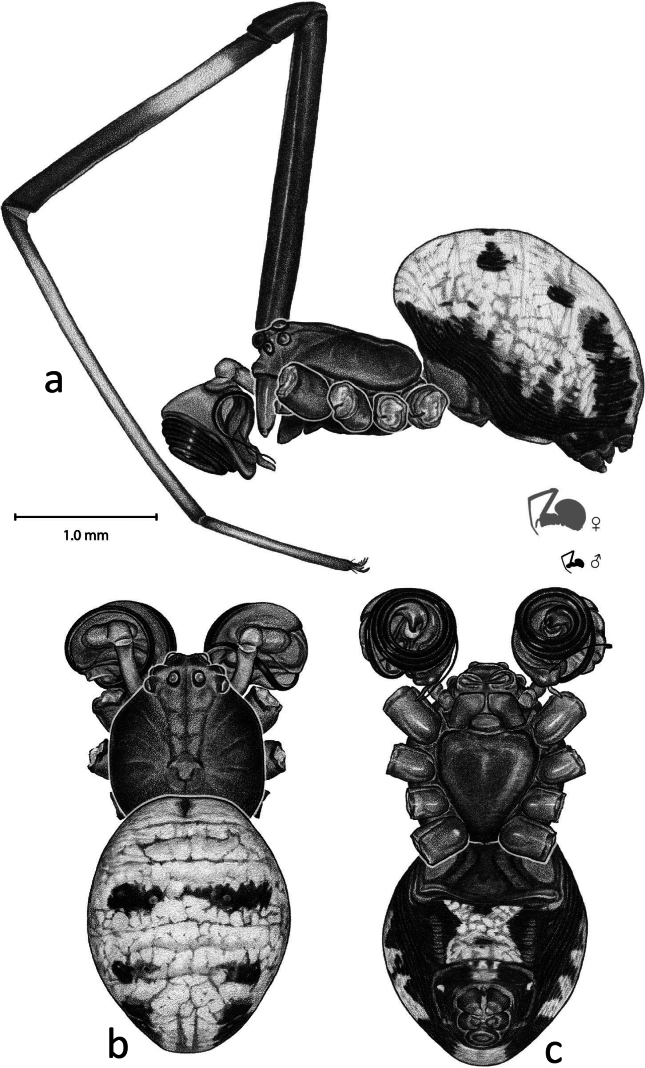
*
Latrodectus lucacha* Miller, Kratzer & Griswold, sp. nov., from Socabaya, Arequipa, Peru (MUSM-ENT 0519563), illustrations of adult male. **A**. Lateral; **B**. Dorsal; **C**. Ventral. Inset, silhouettes of female (gray) and male (black) showing relative size. Scale bar: 1.0 mm.

**Figure 7.**
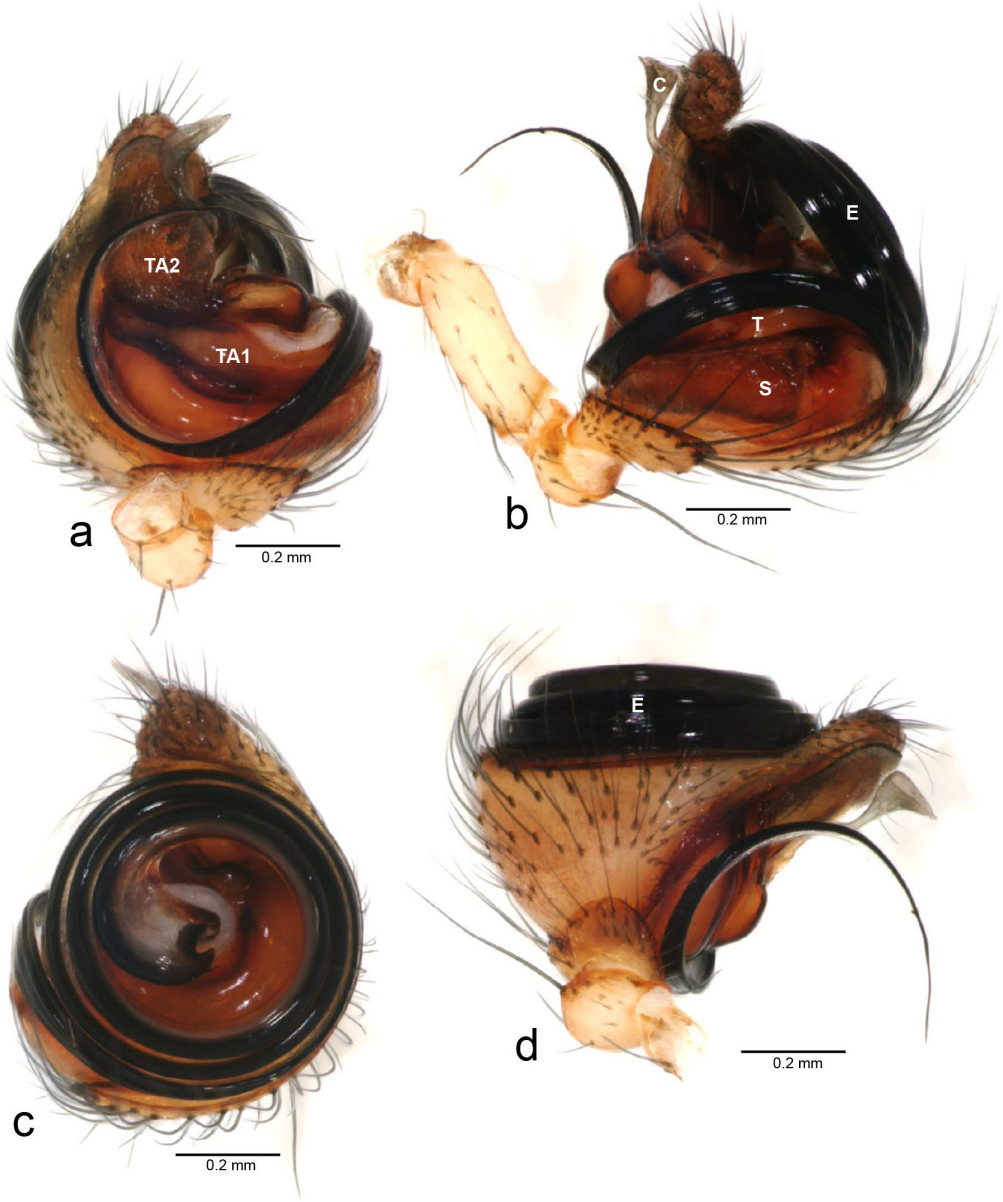
*
Latrodectus lucacha* Miller, Kratzer & Griswold, sp. nov., from Socabaya, Arequipa, Peru (MUSM-ENT 0519563), left male pedipalp. **A**. Prolateral; **B**. Ventral; **C**. Retrolateral; **D**. Dorsal. Abbreviations: C conductor, E embolus, S subtegulum, T tegulum, TA1 tegular apophysis 1, TA2 tegular apophysis 2. Scale bars: 0.2 mm.

Males of most *Latrodectus* species feature contrasting markings on the opisthosoma; in *L. lucacha* sp. nov., the dorsal surface of the opisthosoma is more or less uniform white (Figs 2A–C, 6); *L. thoracicus* is similar but usually with at least some dark markings; only the male of *L. antheratus* similarly has low contrast markings, but is overall dark. Embolus in *L. lucacha* sp. nov. longer than that of either *L. thoracicus* or *L. antheratus*, making ca 3 full spiral turns over retrolateral part of pedipalp (Fig. 7C) before making another nearly full turn over prolateral surface of pedipalp (Fig. 7A). In *L. thoracicus*, the embolus makes ca 2.5 spiral turns over the retrolateral part of the pedipalp before making another half turn over the prolateral surface; in *L. antheratus*, the embolus is even shorter, making ca. 2 spiral turns over the retrolateral part of the pedipalp before continuing another partial turn.

Egg case small (around 7–10 mm diameter), white, spherical, moderately woolly, lacking ornamentation (Fig. 1A, B). It resembles that of *L. antheratus*, which is in about the same size range but tends towards a more pyriform shape. Unlike the spherical to pyriform egg cases of *L. geographicus* (around 6–10 mm), it lacks pompoms loosely attached to the surface that can be lost when handled (Abalos 1962: fig. 3). It is smaller than that of *L. thoracicus*, which is reported to be as large as 21 mm (Abalos 1962: fig. 4) although the ones we observed directly were around 12 mm; *L. quartus* is also reported to be quite large and woolly, not exceeding 17 mm (Abalos 1980) although the ones we observed were around 12 mm. We did not examine egg cases of *L. diaguita*, but they are reported to be quite large (ca. 22 mm) and yellow (Abalos 1980).

#### Description.

***Female*** (holotype from Pachacayo, Junin, Peru): total length 8.1, carapace length 3.3, carapace width 3.1, leg I length 17.0 (femur 5.1, patella 1.6, tibia 3.8, metatarsus 4.9, tarsus 1.6), carapace, sternum, chelicerae and legs all dark brown to black with subtle variation in intensity (Fig. 5). Legs without clear stripes or annulations. Opisthosoma setae mostly long and uniform in length (Fig. 3D). Opisthosoma black with longitudinal red stripe running from just above spinnerets towards middorsal region (Figs 4, 5B). Red ventral opisthosomal marking between epigastric furrow and spinnerets, roughly box-like with distinct lateral notches anterior to centerline (Figs 3A, 5D).

Epigynum: atrium distinct (Fig. 3C). Spermathecae dumbbell-shaped with swollen anterior and posterior lobes connected by a thinner region. Posterior lobes nearly touching, anterior lobes diverging. Copulatory ducts make three loops lateral to the spermathecae, then an arc to the atrium between spermathecae (Figs 2D, 3D–F).

***Male*** (from Pantanos de Villa Om, Lima, Peru): total length 2.8, carapace length 1.2, carapace width 1.1, leg I length 8.3 (femur 2.2, patella 0.6, tibia 2.0, metatarsus 2.4, tarsus 1.1), carapace, sternum, and chelicerae dark brown with some lighter and darker patches (Fig. 2A–C, 6). Basal leg part of legs mostly dark brown, with proximal halves of tibiae, metatarsi and tarsi light tan. Dorsal surface of opisthosoma mostly white, ventral surface darker with marking between epigastric furrow and spinnerets somewhat box-like with distinct lateral notches anterior to centerline.

Pedipalp: subtegulum (S) smooth, mostly occupied by fundus of sperm duct, attached to cymbium via membranous basal haematodocha. Tegulum (T) conducts sperm duct to embolus. Two tegular apophyses (TA1 and TA2) and a flexible conductor (C) arise from tegulum (T); TA1 projects dorsally from near middle of tegulum; TA2 arises distal to TA1, projects distally with rough texture and serrated margins; conductor distinctly swollen distally, shape resembling a bird head (Fig. 7). Embolus (E) with membranous connection to tegulum, with median notch at origin, relatively thick, makes three spiral loops around retrolateral face of bulb, then runs to prolateral face where it makes nearly one spiral loop. Dentiform projection indicating embolic break point visible approaching embolic tip.

#### Variation (6♀/2♂).

Total length 7.5–9.9/2.8–3.3; carapace length 2.9–3.6/1.2; carapace width 2.9–3.2/1.0–1.1. Longitudinal red stripe on opisthosoma of female variable in length running from just above spinnerets towards middorsal region (Figs 4, 5B). Stripe distinctly uneven in width and may run nearly the entire posterior surface of the opisthosoma or be quite short and restricted to area near spinnerets; anterior part of stripe may have a more or less triangular head; sometimes with separate curved transverse stripe, thickest near dorsal center of opisthosoma and tapered towards lateral surface.

#### Distribution.

**Figure 8.**
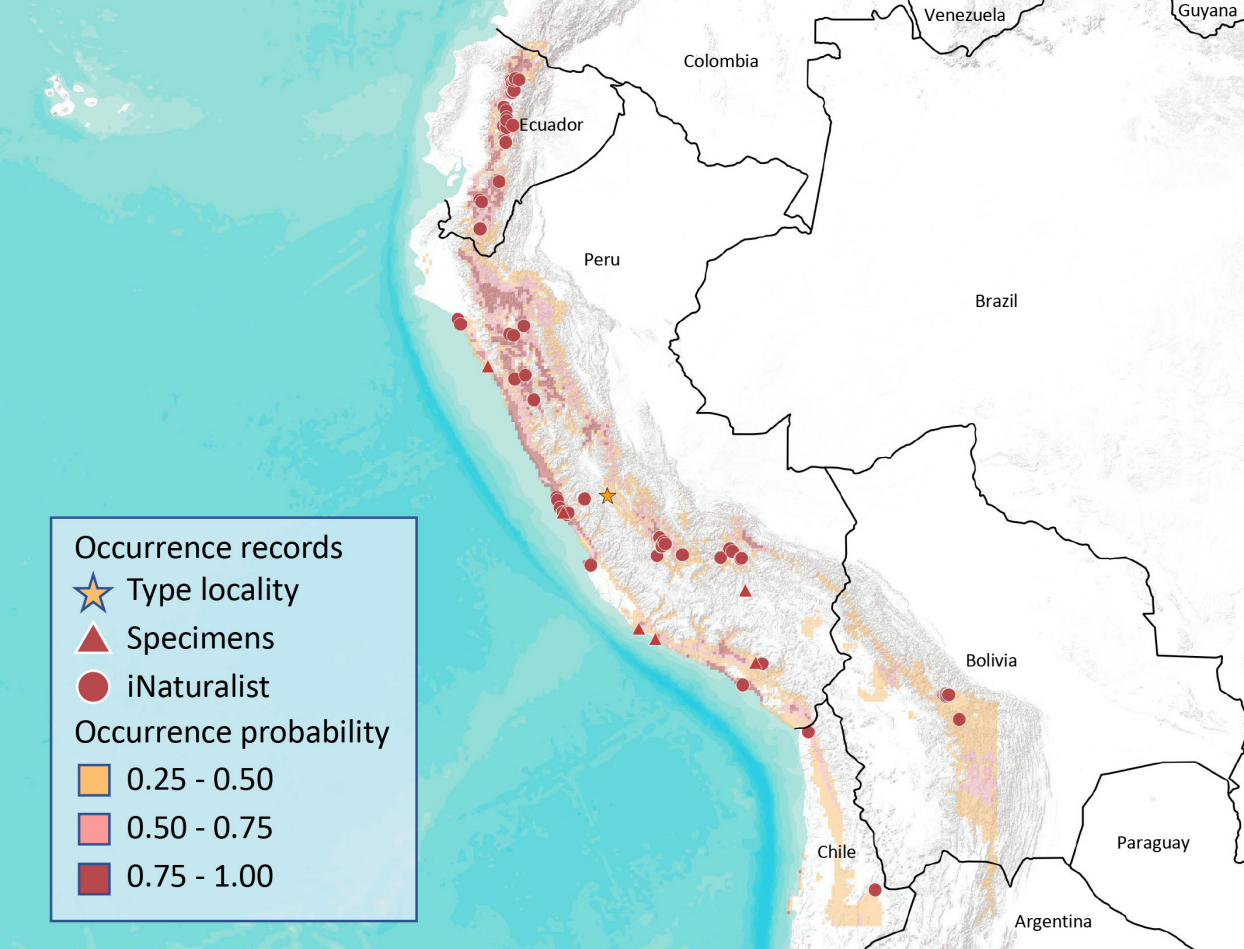
Map of northern South America showing distribution of *Latrodectus lucacha* Miller, Kratzer & Griswold, sp. nov. Specimen records indicated by red triangle, or yellow star for the type locality; records inferred from iNaturalist indicated by red circles; species-distribution model based on all records indicates occurrence probability.

Western Andean slopes and intermontane valleys of Ecuador, Peru, Bolivia, and northern Chile between approximately 200–1500 m elevation with warm mean annual temperatures and moderate precipitation (Figs 8, S2).

#### DNA barcode.

We obtained DNA barcode sequences from the holotype and seven other specimens. Most sequences were 649 bases with two a bit shorter at the 3' end. Barcode sequences are 96.6% identical, with variation detected at 22 positions. Process IDs (the unique record identifier for the Barcode of Life Data Systems) are AWSLL001-26–AWSLL008-26 and are associated with specific records in the material examined section.

## Discussion

Pickard-Cambridge (1902) noted a series of local names by which widow spiders were known around the world. Among these was “lucacha”, the name used in Peru. This is perhaps the first reference in the formal taxonomic literature to the species we describe here.

A forthcoming phylogenetic analysis and taxonomic review of the genus *Latrodectus* found that *L. lucacha* sp. nov. belongs to a South American clade (Miller et al. in prep.). Its close relatives include *L. antheratus* and *L. thoracicus*, but the data indicate a distinct species with no sign of introgression with other species. The phylogenetic study addresses several misidentified specimens and nomenclatural issues from previous studies. For example, we consider the species identified as *L. curacaviensis* in Rueda et al. 2021 to be distinct from *L. curacaviensis* (*sensu stricto*), and this is reflected in our diagnosis. We also accept the synonymy of *L. mirabilis* with *L. thoracicus* proposed by Aguilera et al. (2009). We refer to the currently accepted *L. corallinus*, anticipating that this will become a junior synonym (Miller et al. in prep.).

Occurrence records of *L. lucacha* sp. nov. are based on examined specimens as well as photographic records of live animals in the field. One hundred and twenty-two iNaturalist records could be confidently identified as *L. lucacha* sp. nov. based on coloration, egg-case morphology, and/or geographic location (Fig. 1). When working from photographs contributed to citizen-science communities, the following diagnostic guidance applies. Males and juveniles of *L. lucacha* sp. nov. are often readily distinguishable from other South American species by the extent of white coloration on the opisthosoma (Figs 1A, 2A, 6B). Females typically have transverse red stripes on the opisthosoma, though late-instar females can be nearly entirely black (except for a large red spot just above the spinnerets; Fig. 4). The ventral opisthosoma marking in *L. lucacha* sp. nov. always lacks a bright white bar along the anterior edge, which can easily separate it from *L. thoracicus* (Fig. 3A, 5D). These characters, when used together, are often sufficient to identify *L. lucacha* sp. nov. from live photographs to a reasonable degree of confidence.

Adult females of *L. lucacha* sp. nov. exhibit some degree of color pattern variability, which can overlap with the typical dorsal coloration of *L. thoracicus*. However, because there is currently no evidence to suggest that *L. thoracicus* is present as far north as Ecuador, all dorsal photographs of *Latrodectus* from the Northern Andes with an intermediate color pattern are attributed to the new species.

Data from iNaturalist indicate the presence of this species in Ecuador, Chile, and Bolivia, in addition to the specimen-based records from Peru (Fig. 8). As none of the iNaturalist records we used had a species-level identification, none would have “Research Grade,” and therefore they would not be shared with GBIF.org.

Vink et al. (2011) used records from the published literature and research collections databases as well as their own observations to create a species distribution model of *Latrodectus hasselti* Thorell, 1870. Their objective was to identify areas around the world with environmental conditions favorable to the establishment of this synanthropic species. Luo et al. (2022) followed up with a species distribution model of *L. hasselti* based on GBIF combined with literature records. Sadir and Marske (2021) used both climate and anthropogenic data to create species distribution models for *Latrodectus* species in North America with a focus on the synanthropic species *L. geometricus*. Records were compiled from GBIF and a selection of online communities. Taucare-Ríos et al. (2016) used GBIF data supplemented by a collection of records from literature and museum collections to model the distribution of *Latrodectus geometricus* on a global scale. Wang et al. (2018) explored the relative contributions of citizen-science records versus records from museum and taxonomic literature sources to species distribution models, including *Latrodectus variolus* Walckenaer, 1837 as a case study. Cabrera-Espinosa and Valdez-Mondragón (2021) used data from citizen-science communities (but not iNaturalist) to generate species distribution models for selected *Latrodectus* species in Mexico, then provided a species distribution model to accompany the description of their new species *L. occidentalis* Valdez-Mondragón, 2023 (Valdez-Mondragón and Cabrera-Espinosa 2023).

Widow spiders generate considerable interest on citizen-science platforms. This has produced a library of photographs taken at many times and from many places. Widow-spider species are characteristic enough to permit identification from photographs in many cases. We were able to obtain a much more complete picture of the distribution of our new species by combining museum specimen records with citizen-science observations that we were able to identify. Citizen-science records also reveal species interactions, showing several prey items (Fig. 1C, D) and even parasitoids (Fig. 1B).

Miller et al. (in press) compared and contrasted spider species distribution records from institutional and citizen-science sources. They found that while institutional science data covered more of the smaller (and presumably more cryptic and taxonomically challenging) species, citizen-science data provided more records and better geographic coverage for larger (and presumably more conspicuous or charismatic) species. The lesson we take from this analysis is that while many groups of spiders are undersampled or overlooked in the citizen-science data, aggregated field observations of relatively large-bodied lineages, such as widow spiders, might be capable of generating significant new knowledge.

### Medical importance.

Maguiña Vargas et al. (2017) reviewed clinical aspects of envenomation by spiders in Peru. Among the species of concern, they included *Latrodectus*. Although they apply the name *L. mactans*, a photograph provided (fig. 1b) is consistent with *L. lucacha* sp. nov. They note the local names “lucacha” and “huilca” for this species. They report that the spider is common in rural areas, particularly in some agricultural fields. Accidents usually occur outside during the day in the summer. Envenomation results in a stabbing pain (“dolor punzante”) at the site of the bite, which tends to radiate, followed by muscle contractions and rigidity of the abdominal and thoracic walls. Symptoms last 2–3 days. The spider is not aggressive, and no deaths are known to have resulted.

### More to discover?

An unpublished thesis presented data on *Latrodectus* from the Cuzco region of Peru (Caviedes 2024). That study identified two putative new *Latrodectus* species. In one of these, the female has long (4-looped) copulatory ducts and prominent markings on the dorsal surface of the opisthosoma; as such it is distinct from *L. lucacha* sp. nov. The second species is possibly *L. lucacha* sp. nov. However, the males associated with both species are quite dark and not consistent with *L. lucacha* sp. nov. So, we cannot entirely reconcile the observations reported. But it does appear that further *Latrodectus* diversity awaits discovery in the Andes.

## Supplementary Material

XML Treatment for Latrodectus lucacha

## Additional information

### Artificial Intelligence (AI) use

The authors accept full responsibility for the content of the manuscript, including the disclosure of any use of AI.

Artificial intelligence tools were used to assist with coding, and to check text and data for consistency and technical accuracy. All scientific content and conclusions were developed and verified by the authors.

### Author ORCIDs

J. Miller https://orcid.org/0000-0001-8918-9775

C.A. Kratzer https://orcid.org/0009-0004-8030-4924

C. Griswold https://orcid.org/0009-0009-3277-4880

### Data availability

DNA barcode data are available from boldsystems.org (process IDs AWSLL001-26–AWSLL008-26). Specimens are deposited in the MUSM. Other data that support the findings of this study are available in the main text or Supplementary materials.

## Supplementary materials

Supplementary material 1 Supplementary figure S1 Data type pdf Explanation note Evaluation metrics for species distribution models. https://binary.pensoft.net/file/1666686This dataset is made available under the Open Database License (http://opendatacommons.org/licenses/odbl/1.0/). The Open Database License (ODbL) is a license agreement intended to allow users to freely share, modify, and use this Dataset while maintaining this same freedom for others, provided that the original source and author(s) are credited. Miller Jeremy, Kratzer Chris Alice, Griswold Charles

Supplementary material 2 Supplementary figure S2 Data type pdf Explanation note Species distribution model mean occurrence probability and standard deviation. https://binary.pensoft.net/file/1666687This dataset is made available under the Open Database License (http://opendatacommons.org/licenses/odbl/1.0/). The Open Database License (ODbL) is a license agreement intended to allow users to freely share, modify, and use this Dataset while maintaining this same freedom for others, provided that the original source and author(s) are credited. Miller Jeremy, Kratzer Chris Alice, Griswold Charles

Supplementary material 3 Supplementary table S1 Data type xlsx Explanation note iNaturalist observations identified as *Latrodectus lucacha* Miller, Kratzer & Griswold, sp. nov. Included here are the date, location, credit, license, and identifier of observations. https://binary.pensoft.net/file/1666688This dataset is made available under the Open Database License (http://opendatacommons.org/licenses/odbl/1.0/). The Open Database License (ODbL) is a license agreement intended to allow users to freely share, modify, and use this Dataset while maintaining this same freedom for others, provided that the original source and author(s) are credited. Miller Jeremy, Kratzer Chris Alice, Griswold Charles

## References

[B1] Abalos JW (1962) The egg-sac in the identification of species of *Latrodectus* (black-widow spiders). Psyche 69: 268–270. 10.1155/1962/36967

[B2] Abalos JW (1980) Las arañas del género *Latrodectus* en la Argentina. Obra del Centenario del Museo de La Plata 6: 29–51.

[B3] Abalos JW, Báez EC (1967) The spider genus *Latrodectus* in Santiago del Estero, Argentina. In: Russell FE, Saunders PR (Eds) Animal toxins: a collection of papers presented at the First International Symposium on Animal Toxins, Atlantic City, New Jersey, U.S.A., April 9–11, 1966. Pergamon Press, Oxford, 59–74.

[B4] Agnarsson I (2004) Morphological phylogeny of cobweb spiders and their relatives (Araneae, Araneoidea, Theridiidae). Zoological Journal of the Linnean Society 141: 447–626. 10.1111/j.1096-3642.2004.00120.x

[B5] Agnarsson I, Coddington JA, Knoflach B (2007) The morphology and evolution of cobweb spider male genitalia (Araneae: Theridiidae). Journal of Arachnology 37: 334–395. 10.1636/sh-06-36.1

[B6] Aguilera MA, D’Elía G, Casanueva M (2009) Revalidation of *Latrodectus thoracicus* Nicolet, 1849 (Araneae: Theridiidae): biological and phylogenetic antecedents. Gayana 73: 161–171. 10.4067/s0717-65382009000200001

[B7] Barrett RDH, Hebert PDN (2005) Identifying spiders through DNA barcodes. Canadian Journal of Zoology 83: 481–491. 10.1139/Z05-024

[B8] Bhatnagar RDS, Rempel JG (1962) The structure, function, and postembryonic development of the male and female copulatory organs of the black widow spider *Latrodectus curacaviensis* (Müller). Canadian Journal of Zoology 40: 465–510. 10.1139/z62-043

[B9] Cabrera-Espinosa LA, Valdez-Mondragón A (2021) Distribución y modelaje de nicho ecológico, comentarios biogeográficos y taxonómicos del género de arañas *Latrodectus* (Araneae: Theridiidae) de México. Revista Mexicana de Biodiversidad 92: e923665. 10.22201/ib.20078706e.2021.92.3665

[B10] Caviedes KKC (2024) El género *Latrodectus* Walckenaer, 1805 “viuda negra” (Araneae: Theridiidae) en Cuatro localidades de la région del Cusco. Universidad Nacional de San Antonio Abad del Cusco, 112 pp.

[B11] Fick SE, Hijmans RJ (2017) WorldClim 2: new 1 km spatial resolution climate surfaces for global land areas. International Journal of Climatology 37: 4302–4315. 10.1002/joc.5086

[B12] Folmer O, Black M, Hoeh W, Lutz R, Vrijenhoek R (1994) DNA primers for the amplification of mitochondrial cytochrome c oxidase subunit I from diverse metazoan invertebrates. Molecular Marine Biology and Biotechnology 3: 294–299. 7881515

[B13] Garb JE, González A, Gillespie RG (2004) The black widow spider genus *Latrodectus* (Araneae: Theridiidae): phylogeny and invasion history. Molecular Phylogenetics and Evolution 31: 1127–1142. 10.1016/j.ympev.2003.10.012 15120405

[B14] Gerschman BS, Schiapelli RD (1965) El género *Latrodectus* Walckenaer, 1805 (Araneae: Theridiidae) en la Argentina. Revista de la Sociedad Entomológica Argentina 27: 51–59.

[B15] Hebert PDN, Cywinska A, Ball SL, deWaard JR (2003) Biological identifications through DNA barcodes. Proceedings of the Royal Society of London B 270: 313–321. 10.1098/rspb.2002.2218 PMC1691236 12614582

[B16] Kozub D, Shapoval J, Yatsenko S, Starikh V, Dobarskyi A (2000) Helicon Focus. 7.7.5. Helicon Soft Ltd.

[B17] Leibensperger LB (2016) Herbert Walter Levi (1921–2014) and Lorna Levi (1928–2014). Breviora 551: 1–37. 10.3099/MCZ28.1

[B18] Levi HW (1965) Techniques for the study of spider genitalia. Psyche 72: 153–158. 10.1155/1965/94978

[B19] Levi HW (1983) On the value of genitalic structures and coloration in separating species of widow spiders (*Latrodectus* sp.) (Arachnida: Araneae: Theridiidae). Verhandlungen naturwissenschaften vereins Hamburg (NF) 26: 195–200.

[B20] Levi HW, Levi LR (1962) The genera of the spider family Theridiidae. Bulletin of the Museum of Comparative Zoology, Harvard 127: 1–71.

[B21] Luo Z, Mowery M, Cheng X, Yang Q, Hu J, Andrade MCB (2022) Realized niche shift of an invasive widow spider: drivers and impacts of human activities. Frontiers in Zoology 19: 25. 10.1186/s12983-022-00470-z PMC9617396 36307847

[B22] Maguiña Vargas C, Figueroa Vásquez V, Pulcha Ugarte R (2017) Actualización sobre manejo de araneismo en Perú. La Revista Médica Herediana 28: 200–207.

[B23] Miller JA, Condy C, Kulkarni S, Szűts T, Rivera-Quiroz FA, Kratzer CA, Vink C, Garb JE, Coddington JA, Griswold CE, Knutson VL, Schultz TR, Lovejoy N, Andrade MCB (in prep.) An atlas of widow spiders (Araneae, Theridiidae, *Latrodectus*): phylogeny, biogeography, and integrated taxonomy. Zoological Journal of the Linnean Society.

[B24] Miller JA, van Rijn M, van Helsdingen PJ, Hogeweg L (in press) Global patterns in spider occurrence data: monitoring cryptic fauna in an era of human observations networks and computer vision models. In: Dimitrov D, Hormiga G (Eds) Spider Systematics and Taxonomy. Academic Press.

[B25] Peckham GW, Peckham EG (1886) Genera of the family Attidae: with a partial synonymy. Transactions of the Wisconsin Academy of Sciences, Arts and Letters 6: 255–342, tables I–IV.

[B26] Phillips SJ, Dudík M, Schapire RE (2025) Maxent software for modeling species niches and distributions (version 3.4.4). http://biodiversityinformatics.amnh.org/open_source/maxent/ [Accessed 27 April 2026]

[B27] Pickard-Cambridge FO (1902) On the spiders of the genus *Latrodectus*, Walckenaer. Proceedings of the Zoological Society of London 1902: 247–261.

[B28] Pinter LJ (1967) Species of widow spiders in Northern Argentina (*Latrodectus*: Theridiidae). Psyche 74: 290–298. 10.1155/1967/67142

[B29] R Core Team (2024) R: a language and environment for statistical computing. R foundation for Statistical Computing, Vienna, Austria.

[B30] Rueda A, Lozano D, Muñoz-Charry V, Velásquez-Vélez MI, Amézquita A, Parra D, Realpe E (2021) Phylogeny of the genus *Latrodectus* (Araneae: Theridiidae) and two new species from the dry forests in the Magdalena Valley – Colombia. Species 22: 243–265.

[B31] Sadir M, Marske KA (2021) Urban environments aid invasion of brown widows (Theridiidae: *Latrodectus geometricus*) in North America, constraining regions of overlap and mitigating potential impact on native widows. Frontiers in Ecology and Evolution 9: 757902. 10.3389/fevo.2021.757902

[B32] Taucare-Ríos A, Bizama G, Bustamante RO (2016) Using global and regional species distribution models (SDM) to infer the invasive stage of *Latrodectus geometricus* (Araneae: Theridiidae) in the Americas. Environmental Entomology 45: 1379–1385. 10.1093/ee/nvw118 28028084

[B33] Valdez-Mondragón A, Cabrera-Espinosa LA (2023) Phylogenetic analyses and description of a new species of black widow spider of the genus *Latrodectus* Walckenaer (Araneae, Theridiidae) from Mexico; one or more species? European Journal of Taxonomy 897: 1–56. 10.5852/ejt.2023.897.2293

[B34] Vink CJ, Derraik JGB, Phillips CB, Sirvid PJ (2011) The invasive Australian redback spider, *Latrodectus hasseltii* Thorell 1870 (Araneae: Theridiidae): current and potential distributions, and likely impacts. Biological Invasions 13: 1003–1019. 10.1007/s10530-010-9885-6

[B35] Wang Y, Casajus N, Buddle C, Berteaux D, Larrivée M (2018) Predicting the distribution of poorly documented species, Northern black widow (*Latrodectus variolus*) and Black purse-web spider (*Sphodros niger*), using museum specimens and citizen science data. PLOS ONE 13: e0201094. 10.1371/journal.pone.0201094 PMC6082516 30089136

[B36] Zhang X-Q, Li X-Y, Marusik YM, Yan M-C, Yang R-H, Zhang YX, Bu F-Y, Ding Y, Wang MY, Yao Z-Y (2025) Spider taxonomy: a historical and global perspective. Zoological Research 46: 1387–1395. 10.24272/j.issn.2095-8137.2025.229 PMC12940466 41298294

