# Supplementary figures and images for: Description of a new Andean species of widow spider (Araneae, Theridiidae, Latrodectus)
Source: Zookeys. 2026 Jun 2;1281:49–67. doi: 10.3897/zookeys.1281.185973 (PMC13250612; doi:10.3897/zookeys.1281.185973)

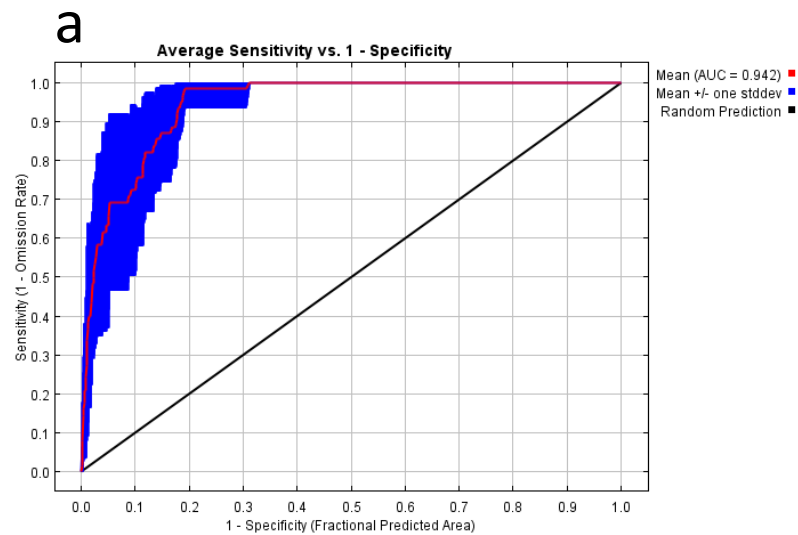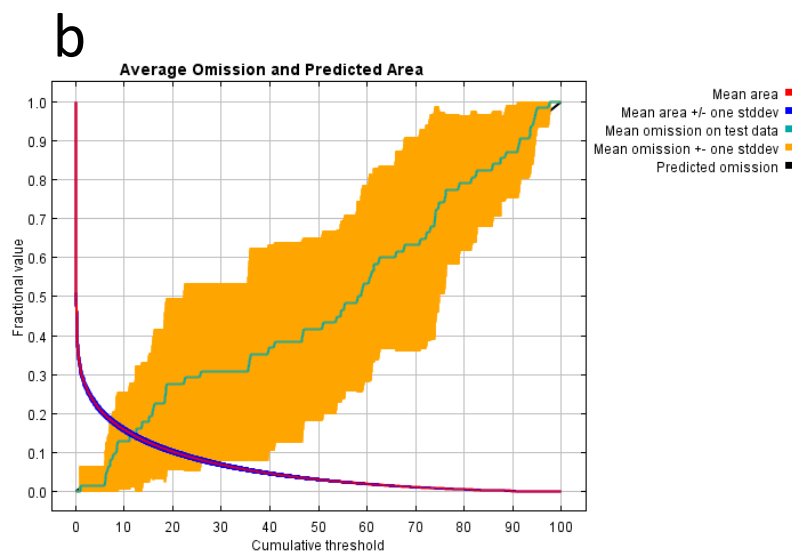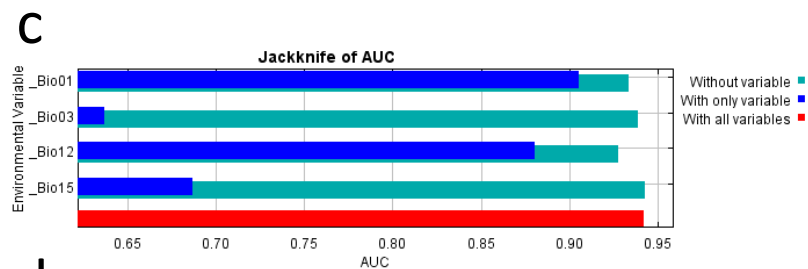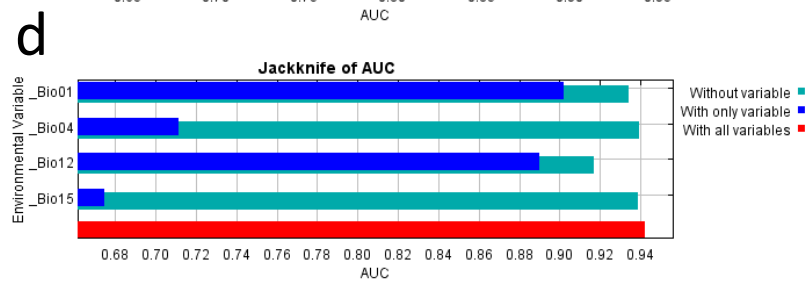

Supplement: Supplementary material 1 — Supplementary figure S1 [file zookeys-1281-049_article-185973__-s001.pdf]

a

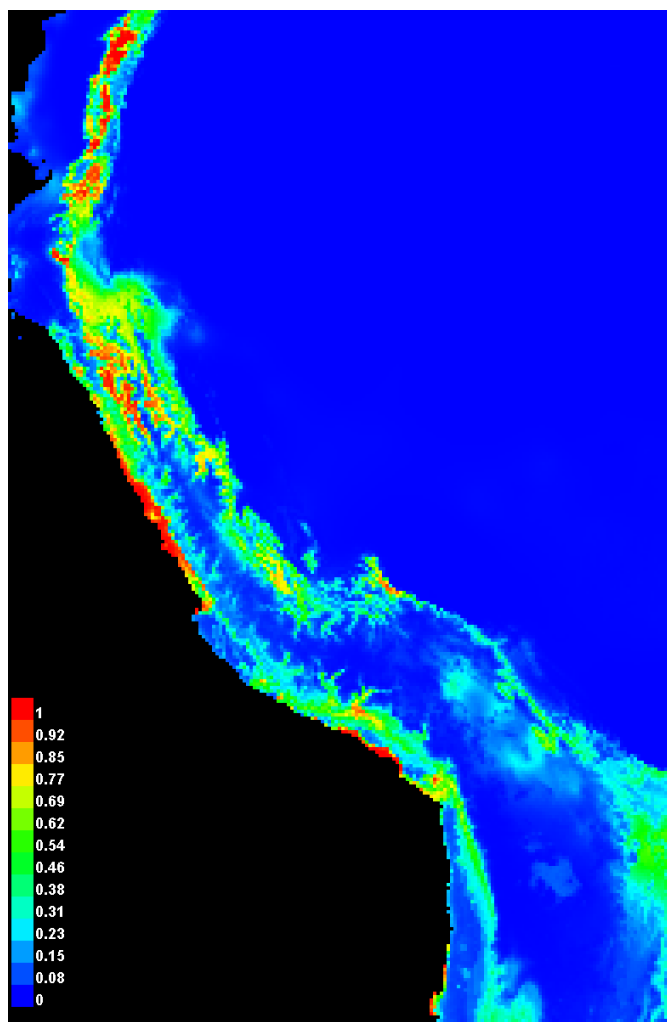

b

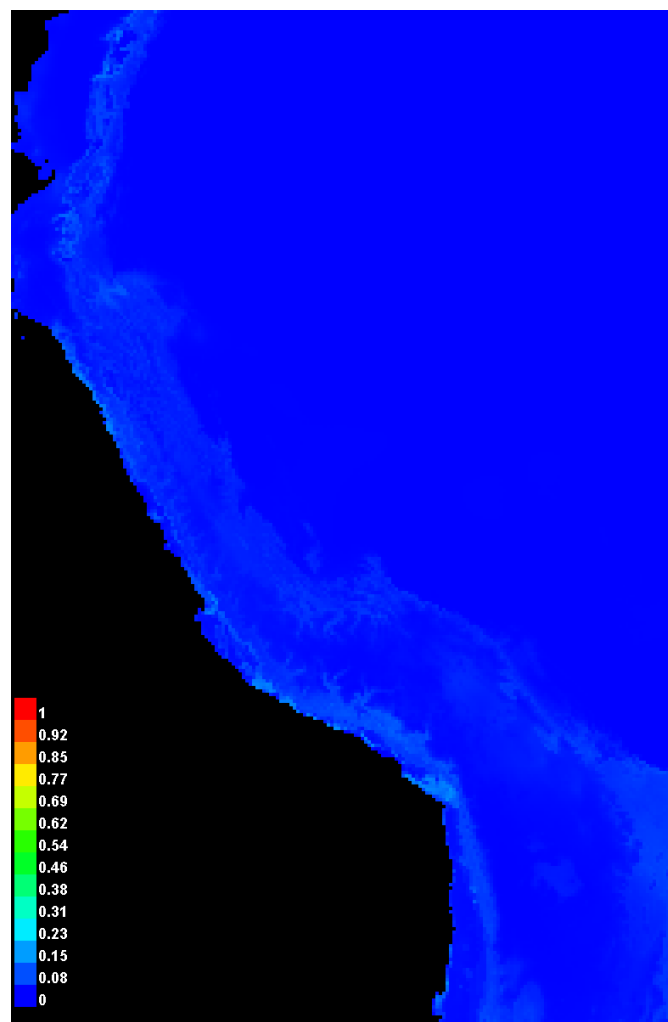

Supplement: Supplementary material 2 — Supplementary figure S2 [file zookeys-1281-049_article-185973__-s002.pdf]
